# The Potential Effect of Na_*v*_1.8 in Autism Spectrum Disorder: Evidence From a Congenital Case With Compound Heterozygous *SCN10A* Mutations

**DOI:** 10.3389/fnmol.2021.709228

**Published:** 2021-07-27

**Authors:** Björn Heinrichs, Baowen Liu, Jin Zhang, Jannis E. Meents, Kim Le, Andelain Erickson, Petra Hautvast, Xiwen Zhu, Ningbo Li, Yi Liu, Marc Spehr, Ute Habel, Markus Rothermel, Barbara Namer, Xianwei Zhang, Angelika Lampert, Guangyou Duan

**Affiliations:** ^1^Institute of Physiology, Uniklinik RWTH Aachen University, Aachen, Germany; ^2^Department of Anesthesiology, The Second Affiliated Hospital, Chongqing Medical University, Chongqing, China; ^3^Department of Anesthesiology, Tongji Hospital, Tongji Medical College, Huazhong University of Science and Technology, Wuhan, China; ^4^Department of Chemosensation, AG Neuromodulation, Institute for Biology II, RWTH Aachen University, Aachen, Germany; ^5^Department of Chemosensation, Institute for Biology II, RWTH Aachen University, Aachen, Germany; ^6^Department of Psychiatry, Psychotherapy and Psychosomatics, Uniklinik RWTH Aachen University, Aachen, Germany; ^7^JARA-BRAIN Institute Brain Structure-Function Relationships: Decoding the Human Brain at Systemic Levels, Forschungszentrum Jülich GmbH and RWTH Aachen University, Aachen, Germany; ^8^Institute for Physiology and Cell Biology, University of Veterinary Medicine, Foundation, Hanover, Germany; ^9^Research Group Neurosciences of the Interdisciplinary Center for Clinical Research (IZKF), Faculty of Medicine, RWTH Aachen University, Aachen, Germany

**Keywords:** *SCN10A*/Nav1.8, autism spectrum disorder, p.I1511M, p.R512^∗^, genetic, mutation

## Abstract

Apart from the most prominent symptoms in Autism spectrum disorder (ASD), namely deficits in social interaction, communication and repetitive behavior, patients often show abnormal sensory reactivity to environmental stimuli. Especially potentially painful stimuli are reported to be experienced in a different way compared to healthy persons. In our present study, we identified an ASD patient carrying compound heterozygous mutations in the voltage-gated sodium channel (VGSC) Na_*v*_1.8, which is preferentially expressed in sensory neurons. We expressed both mutations, p.I1511M and p.R512^∗^, in a heterologous expression system and investigated their biophysical properties using patch-clamp recordings. The results of these experiments reveal that the p.R512^∗^ mutation renders the channel non-functional, while the p.I1511M mutation showed only minor effects on the channel’s function. Behavioral experiments in a Na_*v*_1.8 loss-of-function mouse model additionally revealed that Na_*v*_1.8 may play a role in autism-like symptomatology. Our results present Na_*v*_1.8 as a protein potentially involved in ASD pathophysiology and may therefore offer new insights into the genetic basis of this disease.

## Introduction

Autism spectrum disorder (ASD) represents a complex of neurodevelopmental disorders with multiple symptoms affecting cognitive, social, communication and emotional processes that emerge during infancy and persist throughout life. Recently, it was reported that the prevalence of ASD is about 1.46–2.50%, which has steadily increased over the past decades (Baron-Cohen et al., 2009; Christensen et al., 2016; Xu et al., 2019). The study of ASD has made great strides in the recent years, but the pathophysiological mechanism of ASD remains unclear. It is widely accepted that the causes of ASD include environmental and genetic factors (Sandin et al., 2014). Genetic factors are thought to contribute more substantially than environmental factors and the influence of genetic factors in autism etiology is estimated to be about 50–90% (Sandin et al., 2014; Colvert et al., 2015; Tick et al., 2016; Amaral, 2017). Moreover, there are hundreds of genes associated with ASD, suggesting that autism has a strong, but complex genetic component. A directly causal role of genetic variants, either *de novo* or inherited, in ASD is estimated in 10–30% of the patients, which adds to the strong genetic component in ASD pathophysiology (Vorstman et al., 2017). Therefore, investigating ASD patients with genetic variants may offer new ways in understanding the pathophysiology of the disease.

Numerous studies have revealed changes in expression or function of genes involved in neurodevelopment as the main genetic cause of ASD (Yoon et al., 2020). Excitation/inhibition imbalance is a very popular hypothesis in the pathogenesis of autism (Rubenstein and Merzenich, 2003). It is known that voltage-gated sodium channels (VGSCs) play important roles in excitability of neurons and the mutations of VGSC genes result in a wide range of peripheral and central nervous system disorders (Harkin et al., 2007; Lampert et al., 2010; Kambouris et al., 2017).

Here, we identified a child diagnosed with ASD, and investigated potentially causative genes using a screening sequencing chip for neuropsychiatric disorders. The results revealed two destructive, compound heterozygous variants in *SCN10A*, which were found to be inherited from mother and father, respectively. *SCN10A*/Na_*v*_1.8 belongs to the family of VGSCs and has been considered as a critical factor in the initiation of action potentials in nervous systems (Renganathan et al., 2001), and there is evidence for a role of *SCN10A*/Na_*v*_1.8 in the development of pain disorders (Liu et al., 2014; O’Brien et al., 2019; Li et al., 2020). Interestingly, it has been demonstrated by questionnaire and neuroimaging studies that nociception of ASD patients is commonly disturbed (Riquelme et al., 2016; Failla et al., 2018; Tordjman et al., 2018).

A small part of ASD cases have been reported to be monogenic in origin (de la Torre-Ubieta et al., 2016). Therefore, the mutations of *SCN10A* in our patient may be the best candidate etiology for the ASD phenotype in this case. However, there is no published case of ASD caused by *SCN10A* mutations so far.

In this study, we aimed to explore the potential role of *SCN10A*/Na_*v*_1.8 in the development of ASD. Mutation analysis and whole-cell voltage-clamp were used to investigate the effect of the mutations of *SCN10A* on the biophysical properties of Na_*v*_1.8. Na_*v*_1.8 knockout mice were used as an animal model to explore whether loss of Na_*v*_1.8 results in ASD-like behavioral phenotypes.

## Materials and Methods

### Clinical Investigations

The study regarding this rare case of ASD was approved by the hospital ethics committee of The Second Affiliated Hospital of Chongqing Medical University, and informed consent was obtained from all subjects.

### Genetic Screening and Mutation Analysis

The patient received a genetic testing using a screening chip including 6,110 different target genes related to neurological function by SINOPATH DIAGNOSIS company (Beijing, China) when he was 3 years old. Two heterozygous mutations in *SCN10A* were identified. Because *SCN10A* mutations have not been described to play a role in ASD, we performed a whole genome sequencing for the patient through high-throughput sequencing when he was 4 years old. During the process of the treatment at the age of five, the patient received a whole exome sequencing. At that time, we also collected blood samples and performed *SCN10A* sanger sequencing (primer sequences are shown in Supplementary Table 1) for the patient and his family members. Mutation analysis and function prediction was performed through SIFT, Ployphen-2 and Mutation Taster (Adzhubei et al., 2013; Schwarz et al., 2014; Vaser et al., 2016).

### Mutagenesis

We generated the hNa_*v*_1.8/R512^∗^ and hNa_*v*_1.8/I1511M mutant plasmids in a pIRESpuro3 vector by site-directed mutagenesis using Q5 polymerase (New England Biolabs, Ipswich, MA, United States).

### Cell Culture and Transfection

For electrophysiological examination of the mutations, we used the neuroblastoma cell line ND7/23 (hybrid of mouse neuroblastoma and rat dorsal root ganglion cells). The cells were cultured in Dulbecco’s modified Eagle’s medium (DMEM, Gibco Life Technologies, Carlsbad, CA, United States), supplemented with 4.5 g/L glucose, 10% fetal bovine serum (Gibco Life Technologies, Carlsbad, CA, United States) and 1% penicillin/streptomycin (Sigma Aldrich, St. Louis, MO, United States).

To achieve optimal conditions for transfection, cells were seeded 24–48 h before transfection. Transfection was performed using jetPEI transfection reagent (Polyplus transfection, Illkirch, France). For each transfection a total amount of 1.5 μg DNA was used, consisting of 1.25 μg hNa_*v*_1.8 plasmid (WT, I1511M or R512^∗^) and 0.25 μg GFP. Patch-clamp recordings were performed 24–36 h after transfection.

### Electrophysiology

Transfected ND7/23 cells were recorded using an EPC 10 USB patch-clamp amplifier (HEKA Electronics, Lambrecht, Germany). The sampling rate was 50 kHz with a 10 kHz low-pass Bessel filter. Patch pipettes were pulled using a DMZ pipette puller (Zeitz-Instrumente Vertriebs GmbH, Martinsried, Germany). Pipettes with a tip resistance in the range of 0.9–2.2 MΩ were used. Only green fluorescent cells were chosen for patch-clamp recordings. Experiments were performed at room temperature (22 ± 1°C). The liquid junction potential of 8.7 mV was corrected for prior to the recordings.

The extracellular solution (ECS) contained the following: 140 mM NaCl, 1 mM MgCl_2_, 1 mM CaCl_2_, 10 mM HEPES, 10 mM TEA-Cl and 10 mM glucose. The osmolarity of the ECS was 303 mOsm/L and the pH was adjusted to 7.3 with NaOH. Endogenous sodium currents of ND7/23 cells were inhibited by 500 nM tetrodotoxin (TTX, Tocris Bioscience, Bristol, United Kingdom) added to the bath solution. The intracellular solution (ICS) consisted of 2 mM NaCl, 140 mM CsF, 1 mM EGTA, 10 mM HEPES and 10 mM TEA-Cl. The osmolarity of the ICS was 295–296 mOsm/L and the pH was adjusted to 7.2 with CsOH.

Series resistance compensation was 70–80% and only cells with a series resistance of less than 5.5 MΩ throughout the recordings were included in the analysis. The P/4 procedure was used for online leak current subtraction. After establishing the whole-cell configuration, cells were held at –120 mV and stimulated to 0 mV with a frequency of 0.1 Hz for 5 min to stabilize sodium currents.

#### Voltage Protocols

Voltage dependence of activation was investigated by 40 ms depolarizing pulses between −80 and +70 mV in 10 mV steps every 5 s. For each cell, we determined the reversal potential for sodium (*V*_*rev*_) and calculated the conductance (*G*) at each voltage step (*V*) with the equation $G=\frac{I}{V-V_{r\,e\,v}}$, with *I* being the measured sodium current at the respective voltage step. The conductance-voltage relationship was fitted with a Boltzmann function: $\frac{G}{G_{m\,a\,x}}=G_{m\,i\,n}+\frac{G_{m\,a\,x}-G_{m\,i\,n}}{1+e^{\frac{V_{h\,a\,l\,f}-V_{m}}{k}}}$, with *G*_*min*_ and *G*_*max*_ being the minimum and maximum sodium conductance, *V*_*half*_ the potential of half maximal activation, *V*_*m*_ the membrane potential and *k* the slope factor. In our analysis, *G*_*min*_ was set to 0 and *G*_*max*_ to 1.

Current density was calculated for each cell by dividing the maximum sodium current that occurred during the recordings for the voltage dependence of activation by the cell’s capacitance. The mean current remaining at 30–35 ms after onset of the voltage pulse was measured as the persistent current. Persistent current density was calculated equally to current density.

For each voltage pulse with enough inward current, we also measured the delay from voltage pulse onset to the time point of maximal sodium current (time to peak, TTP). The relationship between TTP and voltage was fitted with an exponential decay function: $t=\left(t_{0}-t_{p\,l\,a\,t\,e\,a\,u}\right)*e^{-\frac{V_{m}}{\tau }}+t_{p\,l\,a\,t\,e\,a\,u}$ (*t* = TTP at the respective voltage, *t*_0_ = TTP at the lowest analyzed voltage step [in our case –10 mV], *t*_*plateau*_ = offset from zero of the decay function, *V*_*m*_ = membrane potential, τ = decay constant).

The decay of inward sodium current after reaching its peak could be fitted with a two-phase exponential decay function: $I=I_{s\,p\,a\,n,f\,a\,s\,t}*e^{-\frac{t}{\tau_{f\,a\,s\,t}}}+I_{s\,p\,a\,n,s\,l\,o\,w}*e^{-\frac{t}{\tau_{s\,l\,o\,w}}}+I_{p\,l\,a\,t\,e\,a\,u}$ (*I* = sodium current at a certain time after current peak, *I*_*span,fast*_ = current range of the fast decay component, *I*_*span,slow*_ = current range of the slow decay component, *I*_*plateau*_ = offset from zero of the decay function, *t* = time after peak of the sodium current, τ_*fast*_ = decay constant of the fast decay component, τ_*slow*_ = decay constant of the slow decay component). This fit was done again for every voltage pulse with enough inward current (in our case starting at –10 mV). The development of the decay constants over the different potentials was afterward plotted and compared.

The voltage dependence of fast inactivation was investigated with a protocol consisting of two pulses. Firstly, a 500 ms pre-pulse to potentials varying between –150 and 0 mV in 10 mV steps was used to drive channels into fast inactivated states. Afterward, the channels remaining in the resting state were activated by a 40 ms test pulse to +40 mV. These two pulses were repeated every 10 s. The measured current (*I*) was normalized to the maximum current in any of the voltage pulses (*I*_*max*_) and plotted against the voltage of the pre-pulse (*V*). The current voltage-relationship was fitted with a Boltzmann function as described above with *G*_*max*_ set to 1.

Steady-state slow inactivation was investigated similar to fast inactivation, but with a three-pulse protocol. At first, a 10 ms pre-pulse to +30 mV activated all channels to act as a reference for the later test pulse. Afterward, the voltage was returned to the holding potential of –120 mV for 300 ms to allow recovery from inactivation. A 30 s inter-pulse to potentials in the range of –110 to 0 mV (in 10 mV steps) then drove the channels into slow inactivated states. Returning to the holding potential for 100 ms after that let channels in fast inactivated states recover from inactivation without inducing a significant recovery from slow inactivation. Finally, a test pulse to +30 mV activated all channels that did not undergo slow inactivation during the inter-pulse. This pulse protocol was repeated every 90 s. The sodium current emerging at the test pulse (*I*_*test*_) was then divided by the current at the pre-pulse (*I*_*pre*_) and plotted against the voltage of the inter-pulse (*V*). The relationship between membrane voltage and the current ratio *I*_*test*_/*I*_*pre*_ was again fitted with a Boltzmann function; *G*_*max*_ was set to 1.

After investigating steady-state slow inactivation we also recorded the time course of the onset of slow inactivation. The voltage protocol was mainly identical as described above for steady-state slow inactivation. This time, we did not vary the voltage of the inter-pulse but its duration. The voltage was always set to 0 mV and the duration varied between 72.9 s and 100 ms (decreased by factor 3 each time). We plotted the current ratio *I*_*test*_/*I*_*pre*_ against the duration of the inter-pulse. The development of this ratio could be fitted with a two-phase exponential decay function similarly to the current decay described above.

### Animals

Homozygous Na_*v*_1.8 knockout mice with C57BL/6 background, generated previously (Akopian et al., 1999; Nassar et al., 2004), were provided by Professor Stephen G. Waxman (Yale University School of Medicine, United States) and maintained in the animal center of Tongji Hospital, Wuhan. A colony of homozygous Na_*v*_1.8 knockout mice was generated from a breeding pair of heterozygous Na_*v*_1.8 knockout mice as described previously (Akopian et al., 1999; Patrick Harty and Waxman, 2007). Genotyping was performed as described previously (Nassar et al., 2004; Gautron et al., 2011). The congenic wild-type mice were provided by the Animal Center of Hubei Province, China. Male and female mice (4–6 month old) were used in this study. All experimental protocols were performed with the approval of the ethical committee of Tongji Hospital, Tongji Medical College, Huazhong University of Science and Technology, and were performed in accordance with the National Institutes of Health guide for the care and use of Laboratory animals (NIH Publications No. 8023, revised 1978).

#### Repetitive Behavioral Tests

Spontaneous grooming test was performed as previously described (Zike et al., 2017). Briefly, mice were individually placed in a transparent chamber without bedding for 10 min to habituate to the testing environment. After that, spontaneous grooming behavior was recorded for 10 min by video camera. Cumulative time spent in spontaneous grooming all body regions was manually recorded and the behavior videotapes were evaluated in a blinded manner.

#### Nest Building Test

Nest building test was performed as previously described (Deacon, 2006). Briefly, the tested mice were transferred to individual cages overnight with food, water and new bedding. A 5 cm × 5 cm pressed cotton square was placed in each cage. The nests formed by the mice were assessed the next morning on a 5-point scale (Deacon, 2006). Nesting scores show the amount of cotton used.

#### Buried-Food Finding Test

Buried-food finding test was performed as previously described (Yang and Crawley, 2009). Briefly, mice were deprived from food and water for one night before the test. On the day of the test, mice were individually placed in a clean cage with fresh bedding, where a food pellet was buried under 2 cm of bedding. The tested mouse was put into the cage and the latency to find the food pellet was recorded manually.

#### Three-Chamber Social Test

Three-chamber social test was performed as previously described (Schmeisser et al., 2012). A social three-compartmented apparatus was used. The apparatus is divided in left, right and center chambers which includes two transparent sliding doors. The steps of the test were as followed:

1. Before sociability testing, the tested mouse (mouse No.1) was allowed to habituate to the testing environment. The tested mouse was placed in the central chamber with the sliding doors closed for 10 min.

2. Then, opening the sliding doors, the mouse No.1 was allowed to explore the sided chambers for 10 min which contained an empty cage respectively.

3. After habituation, an unfamiliar mouse (mouse No.2) with the same genetic background, age and sex was housed in the cage in one of the chambers, while the other chamber contains the same empty cage as mentioned in step 2. During the third 10 min session, the mouse No.1 was placed in the central chamber with the sliding doors opening. The tests were video-recorded, and time spent in each chamber was measured manually. The time spent in contact with the empty cage (object) or with mouse No.2 in the cage were calculated.

4. During the fourth 10 min session, another unfamiliar mouse (mouse No.3) with same background, age and sex was housed in the empty cage in one of the side chambers. Mouse No.1 was placed in the central chamber and allowed to explore for 10 min with the sliding doors opening. The time spent in each chamber, contacting with the familiar mouse No.2 and unfamiliar mouse No.3 were assessed.

### Statistical Analysis

#### Patch-Clamp Data

Two-phase exponential decay fits were performed with Igor Pro Version 6.37 (WaveMetrics Inc., Portland, OR, United States). All other fits were performed with GraphPad Prism 5 (GraphPad Software, San Diego, CA, United States). Figures were created with CorelDRAW X6 (Corel Corporation, Ottawa, Canada). Unless otherwise specified, data are presented as mean ± 95% confidence interval (95% CI) and significance was assumed when *p* < 0.05. For all comparisons, data were first tested for normal distribution via D’Agostino-Pearson test. Whenever normally distributed, comparisons were done by Student’s *t* test, otherwise the Mann-Whitney test was used. A sensitivity power analysis was performed to determine minimal detectable effects (MDE) using G^∗^Power Version 3.1.9.7 (Faul et al., 2007). The following parameters were set to compute Cohen’s *d* as MDE: α = 0.05, 1 – β = 0.95, and sample sizes *n* of the individual experiments. The minimal detectable difference (MDD) was calculated afterwards for each experiment. Results of the power analysis are summarized in Supplementary Table 2.

#### Animal Data

The data of the animal experiment are expressed as mean ± standard error of the mean (SEM). Normal distribution test was performed before the analysis. Data of spontaneous grooming test and nest building test were analyzed by Student’s two tailed, unpaired *t* test. Data of buried-food finding test were analyzed by Mann-Whitney test. Data of three-chamber test were analyzed by two-way repeated ANOVA with a post-hoc paired-samples *t* test.

## Results

### Clinical Picture

The patient is a 6-year-old boy, and he was firstly admitted to the local hospital with intelligence and behavioral development problems when he was 2.5 years old. During the pregnancy, natural delivery and raising the child, there was no obvious abnormality according to his parents. In other family members, no similar cases are reported so far as shown in Table 1. Test of development quotient (Kawabe et al., 2016) was made, and the results showed that he had delayed development in adaptation, gross motor, communicational, cognitive, and social-emotional skills. Magnetic resonance imaging was performed but no obvious abnormality was found. Tests regarding 48 types of congenital metabolic diseases including amino acid, organic acid and fatty acid metabolism were normal, as well as the chromosome karyotypic analysis. We asked the patient’s mother to perform the Kirschner behavioral scale for autism (score: 14) and ABC behavioral (Fung et al., 2016) screening for autism (score: 47), both of which indicated the possibility of the patient being afflicted with ASD. When he was 2 years and 10 months old, the patient was diagnosed with ASD in a local psychiatric center, and he was included in rehabilitation training for children with autism.

**TABLE 1:** Characteristics of autism-spectrum scales for the patient’s family members.

Subject	Sex	Age	Autism-spectrum quotient	ABC behavioral scale	ASD diagnosis?
I-1	Male	66	18	NA	No
I-2	Female	63	15	NA	No
I-3	Male	62	17	NA	No
I-4	Female	58	8	NA	No
II-1	Male	29	11	NA	No
II-2	Female	30	15	NA	No
II-3	Male	35	20	NA	No
II-4	Female	33	21	NA	No
II-5	Male	39	15	NA	No
II-6	Female	38	19	NA	No
II-7	Male	32	21	NA	No
II-8	Female	32	17	NA	No
III-1	Female	5	NA	19	No
III-2	Female	6	NA	20	No
III-3	Male	9	NA	14	No
III-4	Female	7	NA	17	No
Patient	Male	6	NA	47	Yes

*NA, not applicable.*

When he was 5 years old, the patient was sent to another psychiatric center that confirmed the diagnosis. Chinese version of Psychoeducational Profile (Wang et al., 2017b) was tested with a total score of 53, and delayed development in imitation, sensation, gross motor, fine motor, cognitive and language skills were identified. Recently, at the age of six the patient performed an intelligence test using the Chinese version of the Wechsler Preschool and Primary Scale of Intelligence (Huang et al., 2013a) and the total score was less than 45 (language intelligence quotient <41, and operation intelligence quotient = 50), indicating intellectual disability. In addition, as described by his parents, the patient shows decreased sensitivity to painful stimuli in comparison to other children. Quantitative sensory testing (Rolke et al., 2006) was not performed due to the patients’ health status.

### Genetic Screening and Mutation Analysis

**FIGURE 1:**
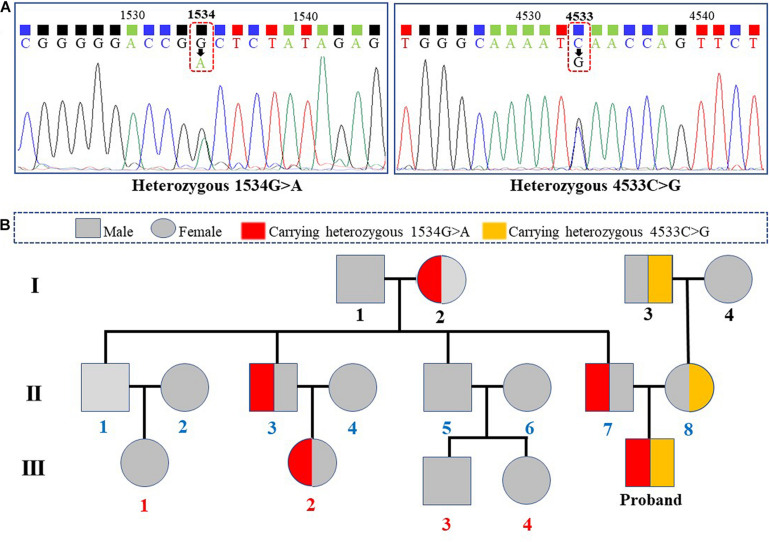
Identification of compound heterozygous *SCN10A* mutations in our patient and inheritance of these mutations in his family. **(A)** Identified heterozygous *SCN10A* mutations in the patient. **(B)** Genetic linkage map for the patient and his family members in three generations.

The screening chip analysis showed that two heterozygous mutations in *SCN10A* (Chr3:38752440, 1534G > A and Chr3:38701963, 4533C > G; NC_000003.12) were identified, and Sanger sequencing was performed to validate the results (Figure 1A). No other specific mutation related to the patient was found in the whole genome sequencing and whole exome sequencing for the patient through high-throughput sequencing. The results of Sanger sequencing for the patient and his family members in three generations are shown in Figure 1B. Only the patient with two compound heterozygous *SCN10A* mutations has been diagnosed with ASD, which conforms to laws of autosomal recessive inheritance.

**FIGURE 2:**
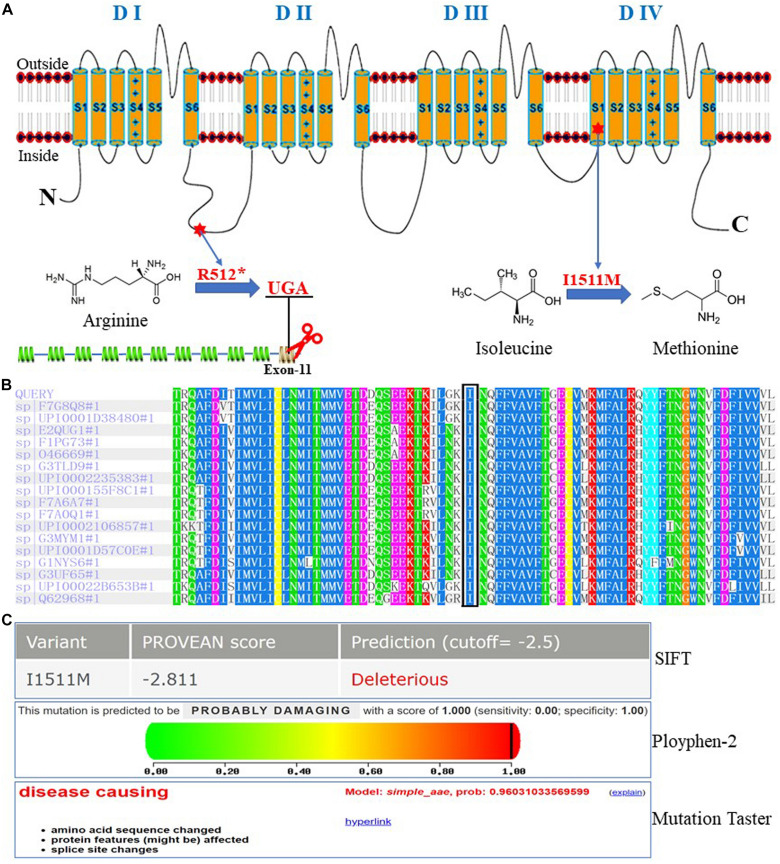
Location, species conservation and predicted effect on protein function of the two identified *SCN10A* mutations. **(A)** The mutation sites for two heterozygous *SCN10A* mutations in Na_*v*_1.8. **(B)** Species conservative analysis for I1511M. **(C)** Function prediction analysis for I1511M.

The *SCN10A* mutation 1534G > A causes the amino acid arginine at 512th site to be substituted by the stop codon UGA, which would prematurely truncate the Na_*v*_1.8 protein translation (Figure 2A). The mutation 4533C > G would cause isoleucine at 1511th site to be substituted by methionine. Species conservative analysis showed that isoleucine was conserved across different species (Figure 2B). All function analysis based on three different prediction tools (SIFT, prediction score −2.811; Ployphen-2, prediction score 1.000; Mutation Taster, prediction score 0.960) conformably showed that p.I1511M can affect the protein function of Na_*v*_1.8 (Figure 2C).

### Truncation Mutation Na_*v*_1.8/R512^∗^ Leads to Loss-of-Function

**FIGURE 3:**
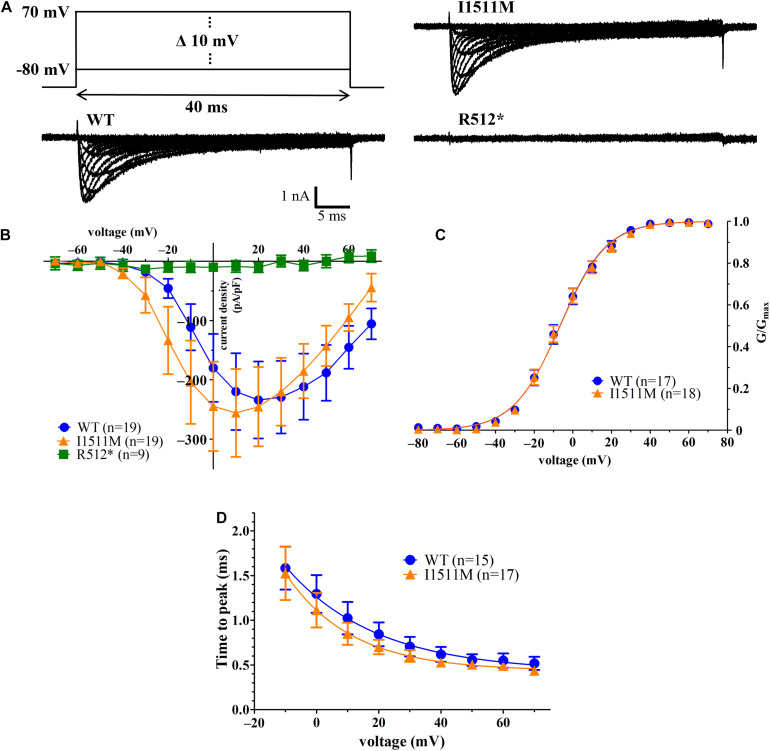
Electrophysiological identification of R512* as a loss-of-function mutation, while I1511M mutated channels produce robust sodium channel activation comparable to WT channels. **(A)** Representative current traces for cells transfected with hNa_*v*_1.8 WT (left trace), I1511M (upper right trace) or R512* (lower right trace). The voltage protocol is shown above the WT trace. **(B)** Current-voltage relationship of hNa_*v*_1.8 WT, I1511M and R512*. **(C)** Conductance-voltage relationship of sodium channel activation of hNa_*v*_1.8 WT and I1511M. **(D)** Development of time to peak values of hNa_*v*_1.8 WT and I1511M over the different voltages. Both data sets were fitted with an exponential decay function.

To test for the functional impact of the identified mutations we performed whole-cell voltage-clamp analysis. ND7/23 cells transfected with the WT hNa_*v*_1.8 plasmid produced fast gating inward sodium current (*n* = 19, Figure 3A). However, cells transfected with hNa_*v*_1.8 R512^∗^ failed to show significant sodium currents in all investigated cells (*n* = 9, Figures 3A,B). Thus, the truncation mutation p.R512^∗^ results in a functional knockout of the hNa_*v*_1.8 channel.

### Activation of Na_*v*_1.8/I1511M Is Unaltered

In contrast to the p.R512^∗^ mutation, the p.I1511M mutation produced inward sodium current with comparable size to the WT channels (Figures 3A,B). The mutated channels activated with the same voltage-dependence as WT (Figure 3C, *V*_*half*_ of activation: –6.6 ± 2.1 mV, *n* = 17 [WT] vs. –6.6 ± 2.0 mV, *n* = 18 [I1511M], *p* = 0.95, slope factor *k* of the Boltzmann function: 11.8 ± 0.5, *n* = 17 [WT] vs. 11.85 ± 0.6, *n* = 18 [I1511M], *p* = 0.80).

The time-to-peak over a range of voltages of each individual cell was fitted with an exponential decay function (Figure 3D), resulting in a decay constant τ representing the voltage dependence of the time-to-peak values. The values of this decay constant were generally comparable for WT and I1511M although they seemed to be very slightly decreased for the mutant channel at the respective voltages.

**FIGURE 4:**
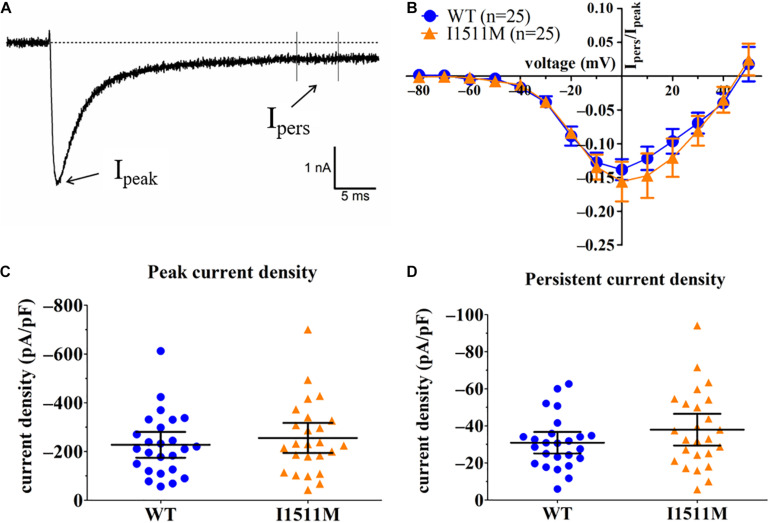
Current density of both peak and persistent current is not affected by the I1511M mutation. **(A)** Representative current trace of a single cell transfected with hNa_*v*_1.8 WT recorded at a voltage step to –10 mV. Arrows indicate time points of peak (I_*peak*_) and persistent (I_*persist.*_) sodium current. **(B)** Plot of current fraction remaining as persistent current (I_*pers*_/I_*peak*_) against the respective voltage. **(C)** Comparison of peak current density of the cells between hNa_*v*_1.8 WT and I1511M. **(D)** Comparison of persistent current density of the cells between hNa_*v*_1.8 WT and I1511M.

Comparison of current density yielded no difference between WT and I1511M (Figure 4C, −227.4 ± 52.7 pA/pF, *n* = 25 [WT] vs. −255.5 ± 61.6 pA/pF, *n* = 25 [I1511M], *p* = 0.57). Both the WT and the mutated channels produced robust persistent current up to 15% of the peak current (Figures 4A,B), which did not differ between WT and Na_*v*_1.8/I1511M either (Figure 4D, −30.9 ± 5.8 pA/pF, *n* = 25 [WT] vs. −37.9 ± 8.6 pA/pF, *n* = 25 [I1511M], *p* = 0.17).

### Na_*v*_1.8/I1511M Fast Inactivation Is Unaffected

**FIGURE 5:**
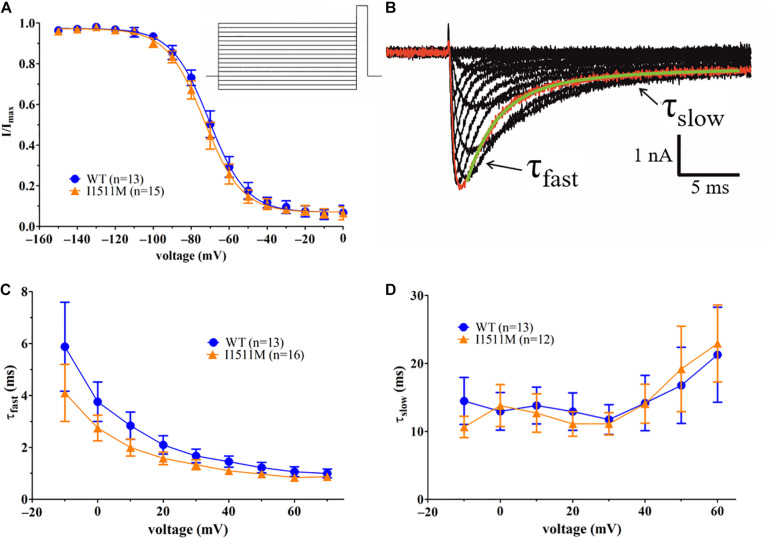
Sodium channel fast inactivation kinetics are comparable for WT and I1511M channels. **(A)** Current-voltage relationship of sodium channel fast inactivation for hNa_*v*_1.8 WT and I1511M. The voltage protocol is shown as inset. **(B)** Example trace for the current decay fit. The trace marked in red was fitted with a two-phase exponential decay function shown in green. The two components of the decay function with their respective decay constant are indicated by arrows. **(C)** Development of τ_*fast*_ values over the different voltages. **(D)** Development of τ_*slow*_ values over the different voltages.

Voltage dependence of sodium channel fast inactivation of WT and I1511M channels was similar for both conditions (Figure 5A, *V*_*half*_ : –71.2 ± 2.1 mV, *n* = 13 [WT] vs. –73.8 ± 2.3 mV, *n* = 15 [I1511M], *p* = 0.08, slope factor *k:* 10.5 ± 1.1, *n* = 13 [WT] vs. 10.6 ± 1.0, *n* = 15 [I1511M], *p* = 0.93). Current decay after activation, when fitted with a two-phase exponential decay (Figure 5B), revealed similar values for τ_*fast*_ as well as τ_*slow*_ (Figures 5C,D).

### Na_*v*_1.8/I1511M Slow Inactivation Is Enhanced

**FIGURE 6:**
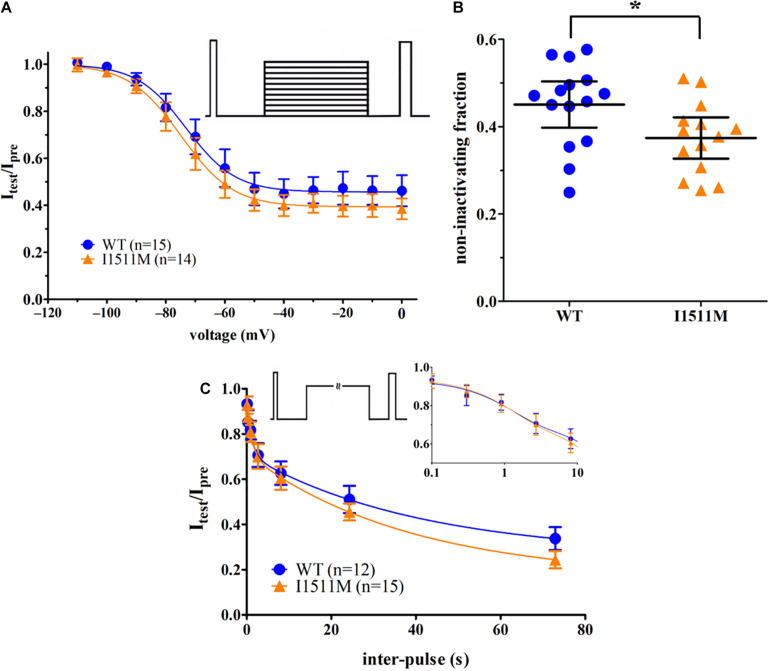
The I1511M mutation shows an enhanced slow inactivation compared to WT channels. **(A)** Voltage dependence of slow inactivation for hNa_*v*_1.8 WT and I1511M. The voltage protocol is shown as inset. **(B)** Comparison of the non-inactivating fraction of the Boltzmann fit shown in panel **(A)** for each cell. **(C)** Onset of slow inactivation. The plot displays the development of current ratio I_*test*_/I_*pre*_ over the different inter-pulse durations. Magnification of shorter inter-pulse durations on a logarithmic x-axis as well as the voltage protocol are shown as inset. *p < 0.05.

We investigated both the voltage dependence of steady-state slow inactivation and the time course of the onset of slow inactivation. Voltage dependence of steady-state slow inactivation was unaffected by the mutation (Figure 6A, –72.0 ± 4.4 mV, *n* = 15 [WT] vs. –73.6 ± 4.3 mV, *n* = 14 [I1511M], *p* = 0.74, slope factor *k*: 7.4 ± 0.7, *n* = 15 [WT] vs. 8.8 ± 1.5, *n* = 14 [I1511M], *p* = 0.21). Interestingly, the fraction of channels resisting slow inactivation at higher voltages (non-inactivating fraction) was reduced for the I1511M mutation (Figure 6B, 0.45 ± 0.05, *n* = 15 [WT] vs. 0.37 ± 0.05, *n* = 14 [I1511M], *p* = 0.03). Thus, at more depolarized voltages, a higher fraction of mutated channels enters the slow inactivated state and therefore fewer channels may remain available for activation.

Onset of slow inactivation was fitted with a two-phase exponential decay function, resulting in similar τ_*fast*_ and τ_*slow*_ (Figure 6C and Supplementary Table 3). However, the non-inactivating fraction of channels was consistently lower for the I1511M mutation at higher inter-pulse durations, which goes well in line with the smaller fraction of channels resisting steady-state slow inactivation at higher potentials (Figures 6A,B).

These results indicate that the mutated channels are more likely to enter the slow inactivated state, mainly at depolarized potentials, which would be reached during channel activity and action potential firing.

### Autism-Like Behavior in Na_*v*_1.8 Knockout Mice

**FIGURE 7:**
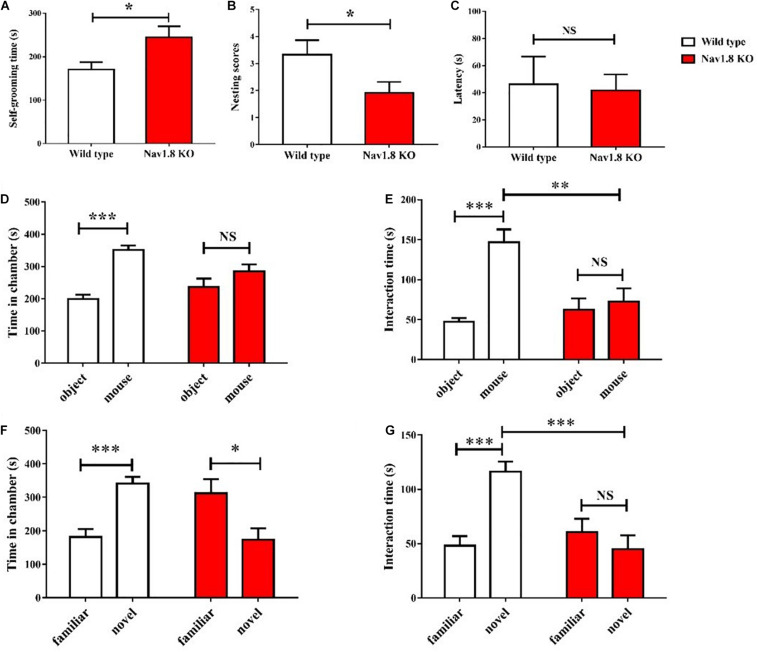
ASD-related behavioral tests in Na_*v*_1.8 knockout mice. **(A)** Self-grooming test. Increased self-grooming time in Na_*v*_1.8 knockout mice. **(B)** Nest building test. Na_*v*_1.8 knockout mice had a poor score in nest building. **(C)** Buried-food finding test. There were no differences in the latency to find a buried food pellet between wild type mice and Na_*v*_1.8 knock-out mice. **(D–G)** Three-chamber social test. Na_*v*_1.8 knockout mice showed a lack of preference for social (mouse) and non-social (object) stimuli and a lack of preference for novel stimuli. *n* = 12 **(A,B)** and *n* = 9 **(C–G)** mice per group. *p < 0.05, **p < 0.01, ***p < 0.001.

To explore the potential role of loss-of-function Na_*v*_1.8 mutations in ASD, we tested for a potential autism-related phenotype of Na_*v*_1.8 knockout mice using behavioral experiments. Increased repetitive behavior is a common symptom in ASD patients. Repetitive behavior in rodents can be analyzed by a spontaneous grooming test (Rabaneda et al., 2014). In the self-grooming test, the time spent in self-grooming of Na_*v*_1.8 knockout mice was higher than that of wild type mice (Figure 7A). Nest building is one of the behaviors in mice, which has been reported to be associated with home-cage social behaviors (Szczypka et al., 2001; Penagarikano et al., 2011). Na_*v*_1.8 knockout mice showed less nest building compared to wild type mice in this task. As shown in Figure 7B, the nesting scores in Na_*v*_1.8 knockout mice were lower than that of wild type mice. The olfactory system is very important for social communication in rodents. Therefore, we examined the latency to find a buried food to rule out olfactory impairment of Na_*v*_1.8 knockout mice before the social interaction test. In the buried-food finding test, the latency to find the food did not differ between wild type mice and Na_*v*_1.8 knockout (Figure 7C).

The three-chamber test is a reliable method for evaluating social behavior in rodents (Yang et al., 2011). The tested mouse was allowed to freely explore and interact with object or mice placed in lateral chambers. The time in each chamber and the time in interaction with each stimulus were calculated. As shown in Figures 7D,E, wild type mice spent more time in the chamber with another mouse than in the chamber with an object, suggesting intact social behavior. In contrast, Na_*v*_1.8 knockout mice showed lack of preference for either the object or the other mouse. Compared with wild type mice, Na_*v*_1.8 knockout mice interacted less with the social stimulus (in this case, the second mouse). In the next step, the object was replaced by a novel, unfamiliar mouse, while the familiar mouse remained accessible as well. Wild type mice showed preferential interaction with the novel mouse than with the familiar mouse. In contrast, Na_*v*_1.8 knockout mice preferred the familiar mouse and spent less time in the chamber with the novel mouse. In addition, Na_*v*_1.8 knockout mice spent similar time interacting with the familiar and the novel mouse (Figure 7G). Compared with wild type mice, Na_*v*_1.8 knockout mice displayed reduced interaction with a novel mouse (Figures 7F,G). These results showed altered social behavior of Na_*v*_1.8 knockout mice.

## Discussion

In the present study, we identified a patient with ASD, which is possibly caused by two novel compound heterozygous *SCN10A* mutations. Through electrophysiological examination, we found that the p.R512^∗^ mutation renders the channel completely non-functional, whereas the p.I1511M mutation showed subtle changes to the channel’s biophysical properties. In addition, behavioral experiments revealed that Na_*v*_1.8 knockout mice present behavioral abnormalities that resemble a typical ASD phenotype.

The most apparent symptoms of ASD comprise deficits in social interaction, communication, and repetitive, stereotypical behavior. Accordingly, these symptoms represent the diagnostic criteria of this group of neurodevelopmental disorders (American Psychiatric Association, 2013). But additionally, a significant fraction of ASD patients also show abnormal reactions to environmental stimuli such as somatosensory input (Tomchek and Dunn, 2007). In fact, hyper- or hyporeactivity to different stimuli can act as predictor for diagnosis and/or severity of ASD (Grzadzinski et al., 2020; Jussila et al., 2020). The social impairment may in part be due to this altered perception of the environment. It has been shown that sensory abnormalities in early life relate to the development of social symptoms in children diagnosed with ASD (Baranek et al., 2018).

The molecular mechanisms underlying these somatosensory abnormalities are associated with distinct peripheral somatosensory neurons, such as low-threshold mechanoreceptors (LTMRs) which are responsible for the perception of innocuous tactile stimuli (Orefice et al., 2016; Orefice, 2020). Apart from the mainly studied myelinated Aβ- and Aδ-LTMRs, there are also unmyelinated LTMRs (C-LTMRs, reviewed in Liljencrantz and Olausson, 2014), that are suggested to play a role both in neuropathic pain (Liljencrantz et al., 2013) and affective or social touch (Loken et al., 2009), showing another connection between sensory perception and social interaction.

There is also growing evidence for an involvement of nociceptive neurons in ASD pathophysiology, as ASD patients often show abnormal reactions to potentially painful stimuli (Tordjman et al., 2009; Klintwall et al., 2011; Moore, 2015). This involvement of nociceptors is well in line with the observations in our patient, who was reported to be less sensitive to painful stimuli. This fits to our hypothesis of a role of Na_*v*_1.8 in the pathogenesis of ASD in the patient, because Na_*v*_1.8 is expressed in nociceptors and is important for their function.

Another link between ASD and Na_*v*_1.8 can be found in the example of *SHANK3*, which is a scaffolding protein that has been found to be responsible for a subset of monogenetic ASD cases (Sheng and Kim, 2000; Moessner et al., 2007). Mutations in *SHANK3* are also linked to another neurodevelopmental disorder, the Phelan-McDermid syndrome (Phelan and McDermid, 2012). This syndrome shows prominent changes in somatosensory responses, namely hyperreactivity to light touch and at the same time hyporeactivity to painful stimuli (Philippe et al., 2008; Sarasua et al., 2014). On a molecular basis, apart from different brain regions, *SHANK3* can be found at presynaptic terminals of somatosensory neurons in the dorsal horn of the spinal cord (Han et al., 2016b; Orefice et al., 2019). Han et al. (2016b) observed a marked reduction of nociceptive behavior in mice with *SHANK3* haploinsufficiency. They report reduced pain responses in models of inflammatory as well as neuropathic pain especially concerning heat hyperalgesia. This effect could be narrowed down to interaction of *SHANK3* with *TRPV1* in neurons positive for Na_*v*_1.8.

VGSCs such as Na_*v*_1.8 are responsible for the fast upstroke of the action potential and are thus an important regulator of neuronal excitability. Neuronal excitability is commonly implicated in pathophysiological theories of ASD. Especially the theory of excitatory/inhibitory imbalance (Rubenstein and Merzenich, 2003) offers a way of potentially explaining ASD symptoms. Alterations of VGSCs could create such an imbalance and therefore cause autistic traits. Accordingly, VGSC isoforms expressed preferentially in the brain, such as Na_*v*_1.1 and Na_*v*_1.2 (Weiss et al., 2003; O’Roak et al., 2011; Sanders et al., 2012), but also Na_*v*_1.7 (Rubinstein et al., 2018), which can be found in nociceptors, were linked to ASD before. Na_*v*_1.8, which is preferentially expressed in the peripheral nervous system, has so far not been associated with ASD. Na_*v*_1.8 was shown to be involved in diseases of the peripheral nervous system, such as chronic pain disorders (Faber et al., 2012; Huang et al., 2013b; Han et al., 2014; Kist et al., 2016), and arrhythmias (Bezzina et al., 2013). The channel’s expression pattern and its associated diseases suggest a mainly peripheral effect of Na_*v*_1.8 and its mutations. Our investigations indicate that there may be a potential link between a loss-of-function of Na_*v*_1.8 and the development of ASD. It is well asserted that loss-of-function in the isoform Na_*v*_1.7, which is also mainly expressed in the peripheral nervous system, causes the disorder congenital insensitivity to pain (CIP) (Cox et al., 2006). This disease is not associated with any autistic symptoms in affected patients. Therefore, one could hypothesize that loss-of-function in Na_*v*_1.8 may affect peripheral neurons in a different way than loss-of-function in Na_*v*_1.7.

Another explanation arises from a potential additional involvement of Na_*v*_1.8 also in the central nervous system. Generally, the channel’s expression seems to be limited to the peripheral nervous system. However, in pathological conditions, the channel was also found in central neurons, offering the possibility of a broader involvement in neurological diseases (Han et al., 2016a). Although these findings were not related to ASD, a role of Na_*v*_1.8 in a dysfunctional brain development cannot be ruled out. Furthermore, recent genetic research revealed variants in *SCN10A* that are linked to epileptic disorders (Kambouris et al., 2017).

The cardinal symptoms of our patient include intellectual disability, reduced verbal skills and hyperactivity. Through next-generation sequencing including 6,110 possibly related genes, we found two heterozygous *SCN10A* mutations (one missense mutation and one terminator mutation). Both mutations were not reported before. We verified the screening results and investigated his parents using Sanger sequencing, which indicated that it is in accordance with the separation of Mendelian law. Both parents carry one of the mutations, and do not show signs of ASD. Additionally, all other investigated family members of our patient do not show autistic symptoms. Thus, the genetic data indicated that the combined occurrence of the screened *SCN10A* mutations may account for the patient’s symptoms. However, *SCN10A* was mostly correlated to pain phenotypes before, questioning the mutations to be responsible for ASD. Thus, we further carried out whole genome sequencing and a whole exome sequencing, but failed to detect another potential mutation site except for the *SCN10A* mutations. ASD can also result from other factors, such as an infection during pregnancy, peri- and postnatal risks or other incidences, such as brain anomalies (Wang et al., 2017a). In the patient of this study there was no report on such occurrences. Nevertheless, we cannot rule out potential additional influences in the generation of the ASD symptoms of this patient.

To investigate possible functional changes introduced by the two mutations, electrophysiological experiments were performed. The results of our examinations reveal that the p.R512^∗^ mutation leads to a complete functional knockout of the channel. This was expected, because the mutation leads to an early truncation of the protein after only the first of four transmembrane domains. All four domains are necessary for a correctly folded and functional sodium channel protein (Catterall, 2000). The truncated protein will most likely not be trafficked to the cell membrane, but rather be degraded rapidly from the membrane of the endoplasmic reticulum. This fits our investigations of the R512^∗^ mutation not producing any measurable sodium current (Figure 3A). Therefore, all Na_*v*_1.8 function in the patient’s neurons arises from channels carrying the p.I1511M mutation. Our detailed patch-clamp experiment regarding this mutation show – if anything – a slight loss-of-function due to an enhanced slow inactivation at voltages relevant for VGSC activity and action potential firing. We conclude that these changes in the channel’s biophysical properties may lead to impaired function of Na_*v*_1.8-expressing neurons and to an altered neuronal development. Interestingly, slow inactivation of Na_*v*_1.8 was shown to be an important phenomenon in the regulation of the firing of peripheral dorsal root ganglion neurons (Choi et al., 2007). Furthermore, research on the isoform Na_*v*_1.7 indicates that already slight changes in slow inactivation kinetics can result in disrupted neuronal excitability and a clinically relevant pain phenotype (Han et al., 2012). From the electrophysiological results, we conclude that the compound heterozygous *SCN10A* mutations that we identified may be a contributing factor to a dysfunctional development of the nervous system and thus could support the development of ASD in the here reported patient. Further studies are needed to consolidate these findings.

Adding to our *in vitro* data from patch-clamp experiments, we investigated Na_*v*_1.8 knockout mice to check for ASD-like behavior. And in fact, we saw several changes in behavior compared to wild type mice that demonstrate autism-like traits in the knockout mice.

Therefore, the possible alteration in neuronal function and development due to the two Na_*v*_1.8 mutations may have led to the deficits that the patient developed, and which resulted in ASD, although more investigations are needed to confirm this hypothesis. Given the recent emergence of translational read-through therapies for nonsense mutations in different diseases (Nagel-Wolfrum et al., 2016; Vossing et al., 2020), our results may offer novel therapeutic strategies for the treatment of ASD in the presented patient as well as maybe in other patients in the future.

However, there are some limitations in this study. Firstly, we verified the function of p.I1511M and p.R512^∗^ mutations *in vitro* only. The *in vivo* effects of these mutations need to be further investigated. The animal model investigated in our study does not directly resemble the genotype of our patient, as the mice were carrying a homozygous knockout of Na_*v*_1.8. Mice carrying the mutations of our patient could be generated to provide a murine model for the mutations’ effects for future investigations. Still, the results of the behavioral experiments underline the possibility of a general effect of Na_*v*_1.8 dysfunction on development of a behavioral phenotype comparable to ASD-typic symptoms.

Secondly, as our electrophysiological recordings of the p.I1511M mutation show only subtle changes in some biophysical characteristics, it is possible that this variant may not result in a physiologically relevant dysfunction of Na_*v*_1.8-expressing neurons by its own. It is also possible that the occurrence of both mutations together in our patient may be only a coincidence and not causative for the disease. However, the mutation might introduce changes to Na_*v*_1.8 function on another level than the investigated channel gating, such as channel trafficking, interaction with additional proteins, posttranslational modifications or even changes to ion concentrations. It is also well conceivable that the variant could have an impact on a different splice variant of Na_*v*_1.8 than we investigated in our study. Differential effects of splice variants on biophysical properties as well as posttranslational modification have been shown e.g., for Na_*v*_1.7 (Chatelier et al., 2008; Choi et al., 2010; Farmer et al., 2012).

Thirdly, we have only found one ASD patient which may relate to *SCN10A* mutations, thus the role of Na_*v*_1.8 in ASD needs to be confirmed in more cases. Until more evidence of a contribution of hNa_*v*_1.8 dysfunction in ASD pathophysiology is available, a causative role of this dysfunction in the development of the disease remains a preliminary hypothesis.

Fourthly, the cellular mechanism underlying the effect of Na_*v*_1.8 on ASD needs to be explored in future studies. Especially deciphering whether Na_*v*_1.8 changes disrupt neuronal development by a central or peripheral mechanism would be an important contribution to the understanding of ASD pathophysiology.

## Conclusion

In conclusion, the combination of our genetic, electrophysiological, and behavioral data suggests that hNa_*v*_1.8 dysfunction due to two compound heterozygous mutations may have contributed to the development of ASD in our patient. Na_*v*_1.8 loss-of-function seems to contribute to autistic symptoms in both humans and mice, offering the possibility of a general role of the channel in the disease’s pathophysiology.

Our work therefore newly presents Na_*v*_1.8 as a protein potentially involved in ASD development and adds to the knowledge about the genetic basis of the disorder.

## Data Availability Statement

The original contributions presented in the study are included in the article/Supplementary Material, further inquiries can be directed to the corresponding author/s.

## Ethics Statement

The animal study was reviewed and approved by the Ethical Committee of Tongji Hospital, Tongji Medical College, Huazhong University of Science and Technology.

## Author Contributions

BH designed, performed, analyzed and interpreted patch clamp experiments, and wrote the manuscript. BL performed behavioral experiments and wrote the manuscript. JZ performed behavioral experiments. JM designed, analyzed and interpreted patch clamp experiments, and revised the manuscript. KL, MS, UH, MR, and BN interpreted the data and revised the manuscript. AE analyzed and interpreted the data and revised the manuscript. PH performed mutagenesis experiments. XZhu performed patch clamp experiments. NL and YL analyzed and interpreted the patient data. XZha conceived the study, analyzed, interpreted and discussed the data. AL conceived the study, analyzed, interpreted and discussed the data, and participated in writing and revised the manuscript. GD conceived the study, interpreted and discussed the data, and participated in writing and revised the manuscript. All authors approved the final manuscript.

## Conflict of Interest

The authors declare that the research was conducted in the absence of any commercial or financial relationships that could be construed as a potential conflict of interest.

## Publisher’s Note

All claims expressed in this article are solely those of the authors and do not necessarily represent those of their affiliated organizations, or those of the publisher, the editors and the reviewers. Any product that may be evaluated in this article, or claim that may be made by its manufacturer, is not guaranteed or endorsed by the publisher.
