# Supplementary material for: The Potential Effect of Nav1.8 in Autism Spectrum Disorder: Evidence From a Congenital Case With Compound Heterozygous SCN10A Mutations
Source: Front Mol Neurosci. 2021 Jul 27;14:709228. doi: 10.3389/fnmol.2021.709228 (PMC8354588; doi:10.3389/fnmol.2021.709228)
Supplement: Supplementary file 1 [file Table_1.docx]

Supplementary Material

*Supplementary Table 1:*

| Primer | Sequences |
| --- | --- |
| *SCN10A*1534-F | GCAGGGAAATGGGAGTAGGT |
| *SCN10A*1534-R | TGGACAGGATGATGGCTAAAG |
| *SCN10A*4533-F | CAGATGCCTGGTTTGTGCTG |
| *SCN10A*4533-R | GGAGGAGTGATTATTCCCTGTCAG |
