# Supplementary material for: The Potential Effect of Nav1.8 in Autism Spectrum Disorder: Evidence From a Congenital Case With Compound Heterozygous SCN10A Mutations
Source: Front Mol Neurosci. 2021 Jul 27;14:709228. doi: 10.3389/fnmol.2021.709228 (PMC8354588; doi:10.3389/fnmol.2021.709228)
Supplement: Supplementary file 2 [file Table_2.docx]

Supplementary Material

*Supplementary Table 2:*

| Value | Sample size *n* | | Minimal detectable effect (Cohen’s *d*) | Pooled Standard deviation | Minimal detectable difference |
| --- | --- | --- | --- | --- | --- |
|  | WT | I1511M |  |  |  |
| **Activation** |  |  |  |  |  |
| V_half_ [mV] | 17 | 18 | 1.26 | 4.00 | 5.03 |
| Slope | 17 | 18 | 1.26 | 1.11 | 1.39 |
| Peak current density [pA/pF] | 25 | 25 | 1.04 | 138.92 | 144.57 |
| Persistent current density [pA/pF] | 25 | 25 | 1.04 | 17.76 | 18.48 |
| **Time to peak** |  |  |  |  |  |
| t_0_ [ms] | 15 | 17 | 1.32 | 0.36 | 0.47 |
| t_plateau_ [ms] | 15 | 17 | 1.32 | 0.08 | 0.10 |
| τ [mV] | 15 | 17 | 1.32 | 6.99 | 9.23 |
| AUC | 15 | 17 | 1.32 | 1.57 | 2.07 |
| **Current decay** |  |  |  |  |  |
| AUC of τ_fast_ | 13 | 16 | 1.40 | 4.68 | 6.54 |
| AUC of τ_slow_ | 13 | 12 | 1.51 | 20.17 | 30.41 |
| **Fast inactivation** |  |  |  |  |  |
| V_half_ [mV] | 13 | 15 | 1.42 | 3.80 | 5.39 |
| Slope | 13 | 15 | 1.42 | 1.83 | 2.60 |
| Offset | 13 | 15 | 1.42 | 0.04 | 0.05 |
| **Slow inactivation** |  |  |  |  |  |
| V_half_ [mV] | 15 | 14 | 1.39 | 7.71 | 10.72 |
| Slope | 15 | 14 | 1.39 | 2.08 | 2.89 |
| Offset | 15 | 14 | 1.39 | 0.09 | 0.12 |
| τ_fast_ [s] | 12 | 14 | 1.48 | 0.87 | 1.29 |
| τ_slow_ [s] | 12 | 14 | 1.48 | 26.51 | 39.21 |
| Plateau | 12 | 14 | 1.48 | 0.14 | 0.20 |
| AUC (onset) | 12 | 15 | 1.45 | 5.06 | 7.35 |
