# Supplementary material for: The Potential Effect of Nav1.8 in Autism Spectrum Disorder: Evidence From a Congenital Case With Compound Heterozygous SCN10A Mutations
Source: Front Mol Neurosci. 2021 Jul 27;14:709228. doi: 10.3389/fnmol.2021.709228 (PMC8354588; doi:10.3389/fnmol.2021.709228)
Supplement: Supplementary file 3 [file Table_3.docx]

**Supplementary Material**

*Supplementary Table 3:*

| Value | WT  (mean ± 95% CI, *n*) | I1511M  (mean ± 95% CI, *n*) | Difference of means (95% CI) | *p* |
| --- | --- | --- | --- | --- |
| **Activation** |  |  |  |  |
| V_half_ [mV] | -6.64 ± 2.10, 17 | -6.56 ± 1.95, 18 | 0.08, -2.68 to 2.84 | 0.9532 |
| Slope | 11.75 ± 0.53, 17 | 11.85 ± 0.58, 18 | 0.10, -0.66 to 0.86 | 0.7951 |
| Peak current density [pA/pF] | -227.4 ± 52.7, 25 | -255.5 ± 61.6, 25 | 28.1, -51.0 to 107.2 | 0.5737 |
| Persistent current density [pA/pF] | -30.93 ± 5.81, 25 | -37.94 ± 8.58, 25 | 7.01, -2.10 to 17.12 | 0.1693 |
| **Time to peak** |  |  |  |  |
| t_0_ [ms] | 1.26 ± 0.20, 15 | 1.10 ± 0.18, 17 | 0.15, -0.11 to 0.41 | 0.2412 |
| t_plateau_ [ms] | 0.41 ± 0.04, 15 | 0.42 ± 0.04, 17 | 0.01, -0.04 to 0.07 | 0.6821 |
| τ [mV] | 28.78 ± 3.88, 15 | 22.20 ± 3.59, 17 | 6.58, 1.52 to 11.63 | **0.0125** |
| AUC | 6.64 ± 0.97, 15 | 5.73 ± 0.72, 17 | 0.91, -0.23 to 2.04 | 0.1129 |
| **Current decay** |  |  |  |  |
| AUC of τ_fast_ | 15.58 ± 3.50, 13 | 11.99 ± 1.88, 16 | 3.59, 0.01 to 7.12 | **0.0496** |
| AUC of τ_slow_ | 98.43 ± 13.07, 13 | 95.93 ± 11.72, 12 | 2.50, -14.21 to 19.20 | 0.7602 |
| **Fast inactivation** |  |  |  |  |
| V_half_ [mV] | -71.23 ± 2.08, 13 | -73.81 ± 2.26, 15 | 2.58, -0.38 to 5.54 | 0.0845 |
| Slope | 10.51 ± 1.13, 13 | 10.57 ± 0.99, 15 | 0.06, -1.36 to 1.49 | 0.9272 |
| Offset | 0.07 ± 0.03, 13 | 0.06 ± 0.02, 15 | 0.002, -0.03 to 0.03 | 0.5804 |
| **Slow inactivation** |  |  |  |  |
| V_half_ [mV] | -71.98 ± 4.43, 15 | -73.55 ± 4.27, 14 | 1.56, -4.32 to 7.44 | 0.7434 |
| Slope | 7.38 ± 0.72, 15 | 8.77 ± 1.54, 14 | 1.39, -0.19 to 2.98 | 0.2136 |
| Offset | 0.45 ± 0.05, 15 | 0.37 ± 0.05, 14 | 0.08, 0.01 to 0.14 | **0.0289** |
| τ_fast_ [s] | 0.98 ± 0.54, 12 | 1.39 ± 0.51, 14 | 0.42, -0.29 to 1.13 | 0.1292 |
| τ_slow_ [s] | 47.30 ± 19.70, 12 | 46.79 ± 12.71, 14 | 0.51, -21.01 to 22.04 | 0.9612 |
| Plateau | 0.23 ± 0.10, 12 | 0.13 ± 0.07, 14 | 0.10, -0.02 to 0.21 | 0.0888 |
| AUC (onset) | 36.25 ± 3.38, 12 | 31.13 ± 2.68, 15 | 5.12, 1.08 to 9.12 | **0.0150** |
